# Control-Oriented and Escape-Oriented Coping: Links to Social Support and Mental Health in Early Adolescents

**DOI:** 10.3390/ejihpe15090172

**Published:** 2025-08-27

**Authors:** Megan Cherewick, Madison R. Davenport, Rinzi Lama, Priscilla Giri, Dikcha Mukhia, Roshan P. Rai, Christina M. Cruz, Michael Matergia

**Affiliations:** 1 Department of Community and Behavioral Health, Colorado School of Public Health, University of Colorado Anschutz Medical Campus, 3001 E 17th Pl, Aurora, CO 80045, USA; madison.davenport@cuanschutz.edu; 2 Department of Anthropology, University of North Bengal, Shiliguri 734014, West Bengal, India; rinzi.lama@nbu.ac.in; 3 Darjeeling Ladenla Road Prerna, Darjeeling 734101, West Bengal, India; priscillagiri22@gmail.com (P.G.); mukhiadikcha@gmail.com (D.M.); rairoshan@gmail.com (R.P.R.); 4 Department of Psychiatry, School of Medicine, University of North Carolina at Chapel Hill, Chapel Hill, NC 27599, USA; christina_cruz@med.unc.edu; 5 School Psychology Program, University of North Carolina at Chapel Hill School of Education, Chapel Hill, NC 27599, USA; 6 Broadleaf Health and Education Alliance, Denver, CO 80202, USA; michael.matergia@broadleafhea.org

**Keywords:** coping, mental health, early adolescence, mental wellbeing, social support

## Abstract

This study examined the factor structure of the Kidcope and its associations with social support, psychological symptoms, and mental wellbeing among early adolescents (ages 10–14) in Darjeeling, India. Confirmatory factor analysis supported a two-factor structure: control-oriented and escape-oriented coping. Multivariable regression and structural equation models indicated escape-oriented coping was associated with higher emotional symptoms (b = 3.19; *p ≤* 0.001) and peer problems (b = 1.43; *p* ≤ 0.003), whereas control-oriented coping was linked with lower conduct problems (b = −1.11; *p* = 0.006), and hyperactivity (b = −1.28; *p* = 0.001). Control-oriented coping also correlated with higher mental wellbeing (b = 11.59; *p* = 0.004), prosocial behavior (b = 0.50; *p* ≤ 0.001), and resilience (b = 4.49; *p* ≤ 0.001). Results suggest control-oriented coping mediates 23% of the total effect of social support on psychological difficulties and 15% on resilience. Findings highlight early adolescence as a sensitive window for strengthening coping skills to protect mental health and enhance wellbeing in high-adversity settings.

## 1. Introduction

Coping refers to cognitive and behavioral strategies individuals use to manage stressors that challenge their personal resources. Originally defined by Lazarus and Folkman (1984), the concept of coping has since been understood as a multifaceted process shaped by the nature of the stressor, socioecological context, developmental or life stage, and cultural norms (Lazarus & Folkman, 1984; Skinner & Wellborn, 2019). Coping strategies involve the development, practice, and mastery of mindsets and skills that enable people to navigate, respond to and adapt to stressors (Ewert et al., 2021; Garcia, 2010). Traditionally, coping has been categorized into two primary domains: problem-focused strategies, aimed at resolving the source of stress, and emotion-focused strategies, which seek to address emotional responses or cognitively reframe the perceived meaning and impact of the stressor (Stanisławski, 2019; Tsaur et al., 2016; Powell et al., 2019). More recent theoretical and empirical work has challenged the assumption that coping strategies are inherently adaptive (e.g., problem-focused) or maladaptive (e.g., emotion-focused) (C. Cheng et al., 2014; Cherewick et al., 2024a; Stanisławski, 2019; Wadsworth et al., 2022). Instead, contemporary frameworks emphasize the dynamic, context-dependent nature of coping, highlighting the importance of coping flexibility, the ability to apply strategies flexibly in alignment with specific situational demands (C. Cheng et al., 2021).

This evolving conceptualization of coping has prompted greater attention to how coping is assessed for specific populations like children and adolescents. Among the tools developed, the Kidcope has emerged as one of the most widely used measures for evaluating coping responses in younger populations (Spirito et al., 1996). Initially proposed with a three-factor structure comprised of avoidant coping, negative coping, and active coping, the Kidcope includes 15 items to assess 10 coping strategies such as distraction, problem-solving, and resignation. Its brevity and ease of administration have supported widespread use across diverse cultural settings. However, despite its adaptability, studies have reported variability in the Kidcope’s factor structures across countries and contexts, prompting calls for context-specific validation and interpretation (Vernberg et al., 1996; Vigna et al., 2010).

Observed variability may reflect differences in stressor type and intensity, cultural norms surrounding emotional expression and self-regulation, and the developmental appropriateness of particular coping strategies. Crucially, the perceived adaptiveness of a coping strategy depends not only on the individual, but also on the broader sociocultural and situational environment. For example, while problem-solving is often considered an adaptive strategy in stable environments, it may be ineffective, distressing, or harmful for youth exposed to chronic stressors such as armed conflict, where the root causes of the stressors experienced are beyond their control. Similarly, certain coping behaviors may be stigmatized or discouraged in specific cultural contexts, limiting their accessibility or utility. These considerations underscore the importance of assessing coping within the lived realities of the population studied. Recent theoretical models therefore emphasize coping flexibility, the capacity to draw on a range of strategies and match them to the demands of a given situation, as a more meaningful indicator of adaptive functioning than reliance on any single coping style (S.-T. Cheng & Chan, 2003; Cherewick et al., 2016a; Langrock et al., 2002).

Although the Kidcope has been widely used to assess coping in children and adolescents, its factor structure has shown inconsistency across contexts. For example, Vernberg et al. (1996) identified a four-factor model among adolescents exposed to hurricanes (blame and anger, wishful thinking, positive coping, social withdrawal), while S.-T. Cheng and Chan (2003) found a two-factor structure (control-oriented, escape-oriented) among adolescents in Hong Kong. Vigna et al. (2010) found a one-factor model best explained coping in African American adolescents following Hurricane Katrina, whereas a three-factor model better fit the data for younger children. Among conflict-affected adolescents in the Democratic Republic of Congo, a four-factor model best fit data on coping, including problem-focused, emotion-focused, faith-based, and resignation coping (Cherewick et al., 2016a). These studies demonstrate how coping and its dimensional structure may shift across populations, stressor profiles, and situational demands, emphasizing the importance of psychometric validation and interpretation in culturally specific settings.

While findings from coping underscore the need to recognize cultural and contextual differences in the effectiveness of coping strategies, converging patterns have been observed, particularly when individuals face similar stressors (S.-T. Cheng & Chan, 2003; Cherewick et al., 2016a; Ernestus et al., 2023; Langrock et al., 2002). Emotion-focused and disengagement strategies that are often perceived as maladaptive have been associated with improved mental health outcomes in high-stress, low-resource environments where individuals have little control over their circumstances (Cherewick et al., 2016a; Vernberg et al., 1996; Vigna et al., 2010). Experiencing protracted and compounding stressors may also influence coping strategy patterns. A study with adolescents experiencing high levels of family and economic stress found these youth possess fewer coping skills, relying more on emotion-focused and behavioral disengagement strategies to manage their stress (Wadsworth & Compas, 2002; Waugh et al., 2020). In such settings, avoidance and distraction may serve protective regulatory functions, challenging the assumption that all disengagement strategies are uniformly harmful. Instead, these findings suggest that the adaptiveness of a specific coping strategy depends on the nature of the stressor and the sociocultural environment in which coping occurs. Mental health prevention programming can be improved by a more nuanced and contextually grounded understanding of coping to identify opportunities to adapt and optimize evidence-based interventions that support mental resilience. Early adolescence (ages 10–14) is a particularly important developmental window for intervention, as youth are forming foundational social and emotional skills that contribute to long-term mental health trajectories (Compas et al., 2017; Crone & Dahl, 2012; Flament et al., 2007; Klasen & Crombag, 2013; McCarthy et al., 2016). In low- and middle-income countries (LMIC) like India, where mental health resources remain scarce, interventions that focus on strengthening protective and promotive factors become particularly crucial (Pradeep et al., 2018).

Studies have established the significant relationship between social support and mental health outcomes in youth, including a previous study in this sample (Cherewick et al., 2024b). Coping is a potential mechanism that may mediate links between social support and mental health outcomes. To meaningfully interpret coping patterns, first, the factor structure of selected measurement tools should be completed within a specific context. To our knowledge, no factor analysis of the Kidcope has been conducted among adolescents in India. This represents a critical gap, especially given the large adolescent population in India and the documented burden of youth mental health issues. Nearly half of Indian adolescents report moderate to severe stress, with academic pressure and family relationships being the most commonly cited stressors (Deb et al., 2015; Kumar & Talwar, 2014; Mathew et al., 2015). Emotion-focused coping strategies such as distraction, avoidance, and seeking social support were used by adolescents, while problem-focused coping was less prevalent (Parikh et al., 2019). Determining the factor structure of the Kidcope for Indian adolescents and exploring its associations with mental health and wellbeing can guide the development of culturally relevant prevention programs for early adolescents in the region.

### Study Objectives

The objectives of the study were to: 1. Identify a suitable factor structure of the Kidcope in a sample of early adolescents from Darjeeling, India; 2. Examine associations between coping dimensions and mental health; 3. Evaluate potential mediation through coping strategies between social support and mental health outcomes.

## 2. Methods

### 2.1. Setting

This study was conducted in four schools situated across urban, peri-urban, and rural areas of the Darjeeling Himalayas, a district in West Bengal, India. The region is characterized by its distinct mountain geography and ethnically diverse population, predominantly comprising Indian citizens of Nepali descent, alongside other ethnic groups and economic migrants. The local economy is primarily sustained by tea production, agriculture, tourism, and military service, with informal trade and seasonal migration being common practices. Low-cost private schools cater to approximately 30–50% of the youth population, and their enrolment continues to rise (Rai, 2017). These schools, which typically enrol around 200 students each, operate with minimal governmental support.

### 2.2. Participants

Participants were recruited from four schools that were selected based on positive collaborations with a local non-governmental organization. Initial meetings were held with school principals to explain the study’s purpose, and Memorandums of Understanding were signed to outline potential risks and benefits associated with participation. Eligible classrooms were identified in coordination with school leadership, and a random selection process was used to determine participating classrooms. Eligibility criteria included: (1) ages 10–14, (2) residency in Darjeeling, India, at the time of the study, (3) enrolment in one of the four participating schools, (4) written consent provided by a caregiver, and (5) verbal assent provided by the adolescent participant. All eligible students in the selected classrooms were invited to participate. Caregivers received a description of the study, including assurances of voluntary participation and the option to withdraw at any time. Informed written consent was obtained from caregivers, defined as the adolescent’s primary guardian at the time of the study. Consent forms were read aloud in Nepali to ensure comprehension, and verbal assent was collected from participants during data collection. Adolescents were informed of their right to ask questions, decline participation, or withdraw from the study at any point. Trained research assistants, certified in human subjects research and ethical conduct, facilitated the consent and assent process. Data collection occurred between 8 September and 31 October 2023.

### 2.3. Survey Administration

Surveys were translated from English into Nepali and back-translated by two professional translators to ensure accuracy. Before implementation, the survey was pilot-tested with a small group of adolescents (N = 10) to assess interpretability. Surveys were administered in private locations on school grounds and lasted approximately 45 min. Research assistants verbally read the survey questions aloud in Nepali while participants recorded their responses on paper surveys provided in either Nepali or English, based on their preference. To reduce bias, the study team held pre-survey meetings with caregivers and adolescents to clarify the study’s purpose, detail potential risks and benefits, and emphasize the confidentiality of responses. Importantly, participants were assured that their involvement would have no impact on their academic grades. To safeguard participant wellbeing, all caregivers and participants were provided contact information for local mental health professionals and the study team. Participants received a small incentive, equivalent to $2 USD, deemed culturally appropriate for the context. The incentive aimed to compensate participants for their time and any transportation costs incurred.

### 2.4. Data Management

Trained research assistants entered survey responses into the secure Research Electronic Data Capture (REDCap) hosted at the University of Colorado Anschutz Medical Campus (Version 13.10.2). Data was de-identified to ensure confidentiality, and de-identified data is available in the Supplementary Data section of this article. The research team adhered to rigorous data management protocols, including maintaining detailed records and conducting weekly or biweekly reflective discussions to evaluate findings and refine processes.

### 2.5. Ethics Approval

The study adhered to ethical guidelines for human subjects research in accordance with the Declaration of Helsinki and was approved by the Colorado Multiple Institutional Review Board (Protocol Code/Date: 23-1421/25 August 2023) and the St. Joseph’s College North Pointe Institutional Ethics Committee. Both participants and their caregivers were informed of their right to withdraw at any time from the study and that their participation was voluntary. Written consent was obtained from caregivers, and verbal assent was collected from adolescent participants prior to data collection. Consent and assent protocols were conducted in Nepali to ensure full comprehension.

### 2.6. Survey Measures

Kidcope. The Kidcope is a 15-item self-report questionnaire designed to evaluate 10 common coping strategies in youth when facing stressful situations (Spirito et al., 1996). Participants were asked to recall a specific recent stressor or ongoing challenge and then indicate how often they used each coping strategy outlined in the measure. Example items include “I tried to fix the problem by thinking of solutions” and “I tried to forget about it.” Participant responses were rated on a four-point Likert scale ranging from 1 (strongly disagree) to 4 (strongly agree). Each item corresponds to one of 10 theoretically derived coping strategies: problem-solving (2 items), social support (2 items), cognitive restructuring (1 item), distraction (2 items), emotional regulation (1 item), emotional outburst (1 item), wishful thinking (2 items), resignation (1 item), self-blame (1-item), blaming others (1 item), and avoidance (2 items). For coping strategies assessed by more than one item, we calculated the mean of the relevant items to generate a coping strategy score. Single-item subscales retained their Likert value. Higher scores indicate greater use of the coping strategy. This is a consistently employed approach in prior studies evaluating the dimensional structure and subscale scoring of the Kidcope in youth (S.-T. Cheng & Chan, 2003; Compas et al., 2017; Mels et al., 2015).

Strengths and Difficulties Questionnaire (SDQ). The SDQ is a 25-item measure designed to assess various domains of mental health and behavioral issues in children aged 11–16 (Goodman, 1999). The SDQ evaluates five subscales: hyperactivity/inattention, peer relationship problems, emotional symptoms, conduct problems, and prosocial behaviors. Example items from the emotional symptoms subscale include “I worry a lot” and “I am easily scared,” while the prosocial subscale includes items such as “I try to be nice to other people. I care about their feelings.” Conduct problems are captured with items like “I often lose my temper” and “I am accused of lying or cheating.” Responses were rated on a three-point Likert scale, including: 0 (not true), 1 (somewhat true), or 2 (certainly true). A total difficulties score is derived by summing the scores of the first four subscales, with higher scores indicating greater psychosocial challenges. In this study sample, the SDQ demonstrated acceptable internal reliability for the total difficulties subscale (alpha = 0.72) and the prosocial behaviors subscale (alpha = 0.67).

The Multidimensional Scale of Perceived Social Support (MSPSS). The MSPSS was developed by Zimet et al. (1988) to measure perceived social support using a 12-item scale to assess three domains: family, friends, and significant others (Zimet et al., 1988). Each domain is represented by four items, scored on a 7-point Likert scale ranging from 1 (strongly disagree) to 7 (strongly agree). Examples of family support items include “My family really tries to help me” and “I get the emotional help and support I need from my family.” For the friends subscale, items include “I can talk about my problems with my friends” and “I have friends with whom I can share my joys and sorrows.” The significant others subscale includes items like “There is a special person who is around when I need them.” The total score on the MSPSS ranges from 12 to 84, with higher scores indicating greater perceived social support. The MSPSS has shown strong reliability and validity in diverse populations. In this study sample, the full scale indicated excellent internal consistency, with a Cronbach’s alpha of 0.85. The internal consistency of each subscale was adequate: friends (alpha = 0.79); family (alpha = 0.79); and significant others (alpha = 0.80).

The Child and Youth Resilience Measure-Revised (CYRM-R). The CYRM-R is a 17-item scale that evaluates two dimensions of resilience: intra/interpersonal resilience and caregiver resilience (Jefferies et al., 2019). Widely applicable across diverse cultural contexts, it has been translated into more than 20 languages (Jefferies et al., 2019). Participants rate each item on a 5-point Likert scale, from 1 (strongly disagree) to 5 (strongly agree). Higher scores represent higher levels of resilience. In this sample, the CYRM-R demonstrated strong reliability, with a Cronbach’s alpha of 0.82.

The WHO-5 Mental Well Being Index (WHO-5). The WHO-5 is a brief scale used to assess subjective wellbeing through five items (Topp et al., 2015). Respondents rate their experiences on a 6-point Likert scale, ranging from 0 (at no time) to 5 (all the time). The raw score ranges from 0 to 25 and can then be multiplied by 4, yielding a final score between 0 and 100, where higher scores indicate better mental wellbeing. A positive screen for clinical depression is indicated by a score below 28, and scores below 40 indicate poor mental wellbeing. Sample items include “I have felt cheerful and in good spirits” and “I have felt calm and relaxed.” In this sample, the WHO-5 demonstrated a Cronbach’s alpha of 0.71.

Additional Measures. Key covariates included in the analysis were age, sex, and school location (urban, peri-urban, or rural).

### 2.7. Sample Size and Power

A priori sample size and power analyses were conducted using G*Power 3.1.9.4 software. An a priori analysis for linear multiple regression was performed with a significance level of α = 0.05, power (1 − β) = 0.95, and a two-tailed test, accounting for seven predictors. The analysis indicated that a minimum of 262 participants was required to detect a small effect size β = 0.05 with 95% power, consistent with prior research recommendations (Bhola et al., 2017; Muthén & Muthén, 2002).

### 2.8. Data Analysis

All statistical analyses were conducted using Stata version 14. Descriptive statistics were computed to summarize demographic and survey data. Statistical significance was assessed at the *p* ≤ 0.001 level for all study results. Means and standard deviations of Kidcope responses were compared by gender and age group using *t*-tests. To evaluate the factor structure of the Kidcope, confirmatory factor analysis (CFA) via structural equation modeling (SEM) was conducted, testing four established factor structures from samples with youth. Model fit was evaluated using established criteria: Comparative Fit Index (CFI) and Tucker–Lewis Index (TLI) ≥ 0.90, root mean square error of approximation (RMSEA) ≤ 0.06, and standardized root mean square residual (SRMR) ≤ 0.08 (Hu & Bentler, 1999). Internal reliability was evaluated using Cronbach’s alpha and McDonald’s omega.

Nested regression models were built to explore the associations between coping dimensions and psychosocial outcomes measured by the SDQ (emotional symptoms, conduct problems, hyperactivity, peer problems), as well as positive mental health outcomes (prosocial behavior, WHO-5 Well-Being Index, and resilience via the CYRM-R). The baseline model (Model 1) adjusted for age and sex. Model 2 included coping dimensions, while Model 3 added social support dimensions (family, friends, and significant others) from the MSPSS. The variance explained by each model was examined using R^2^, and model significance was assessed at each step. Lastly, Structural Equation Models (SEM) were fit to examine pathways from social support (MSPSS), through coping, to SDQ total difficulties and total resilience (CYRM-R). These outcomes were selected to represent dual dimensions of mental health: psychosocial symptoms and psychosocial wellbeing. Both measures are commonly used in low-resource contexts, enabling cross-cultural comparisons.

## 3. Results

### 3.1. Descriptive Statistics

The sample consisted of 274 participants, including 135 females and 139 males, all early adolescents aged 10 to 14 years. The mean age was 12.4 years (SD = 1.3). Participants were recruited from various grade levels: Class 5 (N = 41, 15%), Class 6 (N = 56, 20.4%), Class 7 (N = 52, 19.0%), Class 8 (N = 66, 24.1%), Class 9 (N = 44, 16.1%), and Class 10 (N = 15, 5.1%). In terms of geographic location, 142 participants (51.8%) lived in rural communities, 113 (41.2%) in peri-urban areas, and 19 (6.9%) in urban communities.

### 3.2. Kidcope

First, we completed confirmatory factor analyses to assess the adequacy of previous factor structures found in studies with youth. Factor structures were selected based on the original three-factor model derived in a clinical sample used to create the scale, traditional or well-documented models of coping from studies with youth, and actual factor structures based on previous studies using the Kidcope with youth and that included all 15 items (Table 1). Five models were tested: a three-factor model of avoidant, negative, and active coping (Spirito et al., 1995), a two-factor model of emotional and behavioral coping (Lazarus & Folkman, 1984), a three-factor model of primary control, secondary control, and disengagement coping (Connor-Smith et al., 2000), a two-factor engagement and disengagement coping (Valiente et al., 2009), and a two-factor model including escape- and control-oriented coping (S.-T. Cheng & Chan, 2003). Model fit was evaluated using conventional fit indices, including Bentler’s comparative fit index (CFI); (Bentler, 1990), the Tucker–Lewis index (TLI); (Tucker & Lewis, 1973), the root mean square error of approximation (RMSEA); (Steiger, 1980) and the standardized root mean error (SRMR); (Hu & Bentler, 1999).

**Figure 1:**
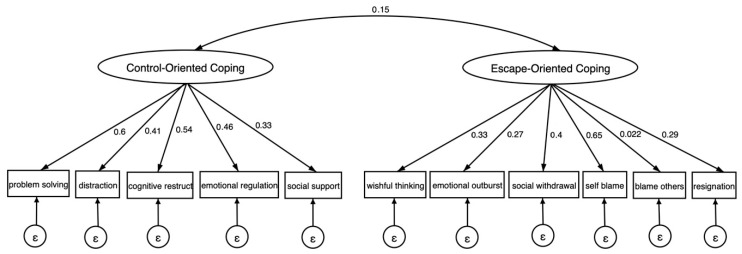
Two-factor confirmatory analysis of the Kidcope (N = 274). Note. Standardized factor loadings: all factor loadings are significant (*p* < 0.05), except for blaming others (*p* = 0.798); CFI: 0.93; TLI: 0.91; SRMR = 0.055; RMSEA = 0.036.

Model fit indices are reported in Table 1. Both three-factor models that were tested failed to converge. The CFA results for the two-factor solution proposed by (S.-T. Cheng & Chan, 2003) best fit these data (CFI = 0.97, TLI = 0.96, RMSEA = 0.032, and SRMR = 0.039. Reliability of the two-factor solution was assessed using both Cronbach’s alpha and McDonald’s omega (Hayes & Coutts, 2020). The reliability coefficients for the control-oriented coping factor (α = 0.62; ω = 0.63) and the escape-oriented coping factor (α = 0.50; ω = 0.49). Although the internal consistency for control- and escape-oriented coping was modest, this is consistent with previous studies using the Kidcope and may reflect the inherently heterogeneous nature of coping behaviors across contexts (Compas et al., 2017). A path diagram illustrating the item loadings grouped by “control-oriented” and “escape-oriented” coping strategies is presented in Figure 1.

### 3.3. Item Responses by Gender and Age

Each item of the Kidcope was analyzed by gender and age group to assess significant differences (Table 2). Female participants scored significantly higher than males on Kidcope Item 3, “I stayed by myself” (t = 2.32; *p* = 0.022). Similarly, females scored higher on Item 7, “I blamed myself for causing the problem” (t = 2.40; *p* = 0.017). Age differences were also evaluated for each item. Older adolescents (aged > 12.5 years) reported higher scores on Kidcope Item 3, “I stayed by myself,” compared to younger participants (t = −3.13; *p* = 0.002). Conversely, younger adolescents (aged ≤ 12.5 years) scored higher on Item 14, “I tried to feel better by spending time with other family and friends,” than their older peers (t = 2.79; *p* = 0.003).

### 3.4. Multivariable Regression Analyses

The results of multivariable regressions on the SDQ constructs are presented in Table 3. Model 1 (M1), which included sex and age, served as the baseline model. Male sex was significantly associated with lower emotional symptoms (b = −1.88; *p* ≤ 0.001). Additionally, each one-year increase in age was linked to higher reported hyperactivity (b = 0.25; *p* = 0.006). M1 explained 16% of the variance in emotional symptoms, 2% in conduct problems, 3% in hyperactivity, and 2% in peer problems. In Model 2 (M2), dimensions of coping were added. M2 accounted for 27% of the variance in emotional symptoms, 9% in conduct problems, 9% in hyperactivity, and 8% in peer problems. Male sex remained significantly associated with lower emotional symptoms in M2 (b = −1.90; *p* ≤ 0.001). Control-oriented coping was significantly associated with lower conduct problems (b = −1.45; *p* < 0.001), lower hyperactivity (b = −1.54; *p* < 0.001), and lower peer problems (b = −1.22; *p* = 0.003).

In Model 3 (M3), the subscales of the MSPSS were added, social support from friends, family, and significant others. M3 explained 28% of the variance in emotional symptoms, 11% in conduct problems, 10% in hyperactivity, and 21% in peer problems. In M3, male sex remained significantly associated with lower emotional symptoms (b = −1.60; *p* ≤ 0.001). Control-oriented coping was associated with lower reported conduct problems (b = −1.11; 0.006), and hyperactivity (b = −1.28; *p* = 0.001). Escape-oriented coping was significantly associated with greater emotional symptoms (b = 3.19; *p* ≤ 0.001) and more peer problems (b = 1.43; *p* = 0.003). Social support from friends was associated with fewer peer problems (b = −0.09; *p* ≤ 0.001), and social support from family was associated with lower levels of peer problems (b = −0.08; *p* = 0.004).

Table 4 presents results of the multivariable regressions on mental wellbeing and resilience outcomes. In Model 1 (M1), 3% of the variance in mental wellbeing, 2% in prosocial behavior, and 6% in resilience were explained. Each year increase in age was associated with lower prosocial behavior (b = −0.21; *p* = 0.012) and lower resilience (b = −1.53; *p* ≤ 0.001). Model 2 (M2) explained 14% of the variance in mental wellbeing, 17% in prosocial behavior, and 27% in resilience. In M2, age was no longer significantly associated with prosocial behavior but remained a significant predictor for resilience (b = −1.12; *p* < 0.001). Control-oriented coping was significantly associated with higher mental wellbeing (b = 22.66; *p* < 0.001), prosocial behavior (b = 1.94; *p* < 0.001), and resilience (b = 11.34; *p* < 0.001). Escape-oriented coping was associated with higher prosocial behavior (b = 0.87; *p* = 0.046). Control-oriented coping was also positively associated with resilience (b = 11.34; *p* < 0.001).

Model 3 (M3) explained 29% of the variance in mental wellbeing, 25% in prosocial behavior, and 58% in resilience. In M3, male sex was significantly associated with higher mental wellbeing (b = 5.89; *p* = 0.012). Coping oriented coping was associated with high mental wellbeing (b = 11.59; *p* = 0.004), prosocial behavior (b = 0.50; *p* = 0.001), and resilience (b = 4.49; *p* < 0.001). Escape-oriented coping was associated with higher prosocial behavior (b = 0.47; *p* = 0.019). Social support from friends was positively associated with mental wellbeing (b = 0.75; *p* = 0.007) and resilience (b = 0.35; *p* ≤ 0.001). Social support from family was linked to higher mental wellbeing (b = 0.85; *p* = 0.004), prosocial behavior (b = 0.08; *p* = 0.004), and resilience (b = 0.77; *p* ≤ 0.001). Social support from significant others was positively associated with mental wellbeing (b = 0.81; *p* = 0.003), and resilience (b = 0.25; *p* = 0.006).

### 3.5. Structural Equation Models

**Figure 2:**
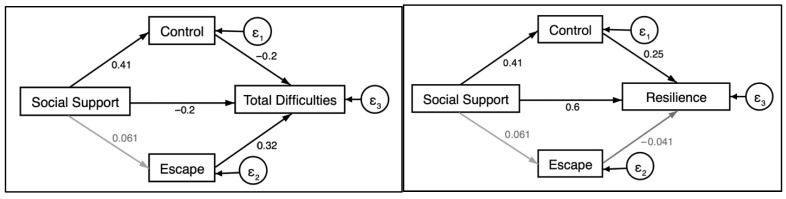
Structural path coefficients to mental health and wellbeing outcomes. Note: Standardized path coefficients displayed. Black lines indicate paths with *p* ≤ 0.05 significance; gray paths are non-significant.

We completed further analysis to explore potential mediating effects by examining the proportion of the total effect of social support on psychological difficulties and resilience, potentially mediated by escape and control-oriented coping (Figure 2). Table 5 displays results that indicate that for total psychological difficulties, 23% of the total effect of social support was mediated by control-oriented coping (*p* ≤ 0.046). Escape-oriented coping was not significantly associated with total social support in the structural model and, therefore, did not mediate this relationship. For total resilience, control-oriented coping accounted for 15% of the total effect of social support on resilience (*p* ≤ 0.0001). Being escape-oriented was not significantly associated with social support or resilience in the structural model.

## 4. Discussion

The current study aimed to: (1) evaluate the factor structure of the Kidcope coping inventory and examine associations between coping, perceived social support, and mental health outcomes in a sample of early adolescents in Darjeeling, India. We found support for a two-factor structure of coping (control-oriented and escape-oriented) consistent with S.-T. Cheng and Chan (2003). This two-factor cooping structure maps closely with other factor models in the literature. Specifically, ‘control-oriented’ coping strategies in our sample, such as problem-solving, seeking social support, and distraction, align with voluntary, engagement-based coping defined by Compas et al. (2017), whereas ‘escape-oriented’ strategies reflect avoidant or disengagement responses such as wishful thinking and withdrawal. These distinctions also intersect with Skinner et al. (2003) function typology of coping, in which coping strategies vary not only in behavioral form but in their underlying regulatory goals (e.g., seeking competence vs. avoiding threat).

Regression results indicate that these coping dimensions were significantly associated with both psychosocial difficulties and positive mental health outcomes. Importantly, control-oriented coping emerged as a key protective and promotive mechanism, with potential mediated effects between perceived social support and mental health outcomes. These findings underscore the role of supportive relationships in fostering adaptive coping in early adolescence. Our results must be interpreted within the broader context of Darjeeling, where our previous quantitative and qualitative research identified dynamic social and structural stressors shaping youth coping. For example, adolescents in this setting frequently report limited opportunities for social engagement, strong academic pressures, and fragmented or migrating family structures that constrain access to social support (Cherewick et al., 2024b). A well-documented limitation of the Kidcope is the variability in its factor structure and psychometric properties across different countries and contexts, such as in contexts of natural disaster and conflict (S.-T. Cheng & Chan, 2003; Vernberg et al., 1996; Vigna et al., 2010). Scholars have suggested that problem-focused coping may be useful when a stressor is manageable, whereas avoidance strategies may be beneficial when stressors are outside of an individual’s control (Littleton et al., 2007).

Multivariable regression analyses demonstrated that control-oriented coping (including problem-solving, cognitive restructuring, social support seeking, emotional regulation, and distraction), was consistently associated with lower mental health difficulties and higher mental wellbeing and resilience. These associations suggest that early adolescents may be developing and applying goal-directed coping strategies, especially in contexts where at least some stressors (e.g., schoolwork) are perceived as controllable.

Notably, distraction strategies, which are often ambiguously categorized in the coping literature, loaded on the control-oriented coping factor in this sample. This suggests that in this context, distraction may function as an active regulatory strategy rather than a passive form of avoidance. These findings are consistent with prior research indicating that distraction can serve as an adaptive mechanism for short-term emotional regulation, particularly under high-stress conditions when immediate problem-solving is not feasible (Cherewick et al., 2016b; Compas et al., 2017). In Darjeeling, sharp increases in academic demands during early adolescence can replace time for play, peer engagement, and emotional processing. Within high-stress academic environments, distraction may enable youth to down-regulate acute emotional distress while preserving cognitive resources (Donaldson et al., 2000).

Unexpectedly, our models (M2 and M3) revealed a positive association between escape-oriented coping and prosocial behavior. Escape-oriented strategies, particularly wishful thinking, may be an example of a disengagement strategy that is often practiced in social environments (faith-based institutions). Religious and spiritual practice holds deep cultural significance in Darjeeling, where Hinduism, Buddhism, and Christianity coexist and shape community values, daily rituals, and coping norms across its ethnically diverse population. While escape-oriented strategies are typically conceptualized as maladaptive, these findings point to a more nuanced relationship. It is possible that certain escape-oriented strategies allow youth to delay emotional reactivity in ways that maintain interpersonal harmony, particularly in the collectivist cultural context of Darjeeling, where social cohesion and family duty are emphasized (Trommsdorff, 2012). In this way, wishful thinking may function as a socially sanctioned form of emotional coping, helping adolescents to remain outwardly prosocial even when experiencing internal distress. Interpreted this way, findings from this study align with others concluding that distraction might facilitate social functioning rather than hinder it (Cherewick et al., 2015; Edlynn et al., 2008). Alternatively, this pattern may reflect a coping behavior mismatch: youth who report more prosocial behavior may still struggle internally with stress, leading them to rely on escape-oriented strategies. This could reflect an “invisible distress phenomenon”, where behavioral adaptation coexists with internal dysregulation not fully captured by conventional measures of internalizing and externalizing symptoms.

In this study, social support was associated with improved mental wellbeing, prosocial, and resilience outcomes. Prior findings from the same sample and other contexts show that social support plays both promotive (directly enhancing wellbeing) and protective (buffering the impact of stressors) roles in adolescent mental health (Cherewick et al., 2024b; Chu et al., 2010; Renwick et al., 2022). One study among Indian youth found that seeking social support mediated the relationship between academic stress and suicidal ideation, emphasizing the role of support networks in stress mitigation (Khan et al., 2016).

Results from structural equation models in the current study found that control-oriented coping partially mediated the effect of social support on both difficulties (20%) and resilience (14%), suggesting that supportive relationships may enhance adolescents’ capacity to respond adaptively to stress. These findings reinforce the value of integrated intervention approaches that not only promote social-emotional learning but also provide structured opportunities for peer connection and support. By equipping adolescents with a repertoire of coping strategies and the relational scaffolding to use them, prevention programs can foster flexible, context-sensitive responses to stressors across developmental transitions.

Limitations. This study includes several limitations. The cross-sectional design precludes causal inference. Longitudinal designs are needed to address coping strategies and social support across development. The generalizability of the findings is limited due to the cross-sectional nature of the study, which is specific to the context of Darjeeling, India. While our CFA supported a control- and escape-oriented coping structure, some subscales had modest internal consistency, and cultural adaptation or alternative response formats may improve measurement reliability. Measurement bias could have influenced results. The Kidcope factors demonstrated modest internal consistency, which may reflect measurement limitations inherent to brief scales like the Kidcope, particularly in diverse, non-clinical populations. Lower internal consistency may also reflect conceptual breadth within latent factors rather than measurement error, underscoring the need for additional items and alternative measurement strategies. Future studies should prioritize further cross-cultural validation of the measure in similar contexts. Response bias could have also influenced the results, as adolescents may underreport or overreport their coping experiences based on social desirability or perceived expectations regarding the coping strategies assessed.

## 5. Conclusions

The study conducted with early adolescents in Darjeeling, India, provides support for a two-factor structure of the Kidcope that distinguishes between control-oriented and escape-oriented coping strategies. Importantly, control-oriented coping was consistently associated with fewer psychological difficulties and stronger levels of mental wellbeing and resilience. In contrast, escape-oriented coping was linked to elevated emotional symptoms and poorer psychosocial functioning. These results highlight the crucial role of early adolescence as a formative period for mental health, underscoring the urgent need for targeted prevention programs. These programs should strengthen youth coping repertoires that emphasize active strategies reinforced through peer and family support. Intervening during this period can set the foundation for mental wellbeing, especially in high-adversity contexts where stressors are frequent and compounding. Equipping youth not only with the tools to cope with stress but also with the supportive environments that enable those tools to be used effectively is essential for building mental health at both the individual and community levels.

## Figures and Tables

**Table 1:** Model fit indices for confirmatory factor analysis based on previous factor structures in studies with youth.

Study	Number of Factors	*χ* ^2^	(df)	*p*	RMSEA	SRMR	CFI	TLI
Spirito et al. (1995)	3	-	-	-	-	-	-	-
Lazarus and Folkman (1984)	2	210.77	89	<0.001	0.071	0.079	0.61	0.54
Connor-Smith et al. (2000)	3	-	-	-	-	-	-	-
Valiente et al. (2009)	2	176.46	88	<0.001	0.061	0.070	0.72	0.66
S.-T. Cheng and Chan (2003)	2	54.29	40	<0.001	0.036	0.055	0.93	0.91

Note: *χ*^2^ = Chi-square test statistic; (df) = degrees of freedom; *p* = *p*-value; RMSEA = root mean square error of approximation (Steiger, 1980); CFI = Bentler’s comparative fit index (Bentler, 1990); TLI = Tucker–Lewis index (Tucker & Lewis, 1973); SRMR = standardized root mean error (Hu & Bentler, 1999).

**Table 2:** Mean responses to Kidcope items by gender and age.

	Male N = 139	Female N = 135			Ages 10 ≤ 12.5	Ages >12.5–14			Total N = 274
Kidcope Items	Mean (SE)	Mean (SE)	t	*p*	Mean (SE)	Mean (SE)	t	*p*	Mean (SD)
**Escape-Oriented**									
*Social Withdrawal*									
I stayed by myself	2.52 (0.10)	2.84 (0.10)	2.31	0.022 *	2.77 (0.10)	2.89 (0.09)	−3.13	0.002 **	2.68 (0.07)
I kept quiet about the problem	2.76 (0.08)	2.70 (0.09)	−0.41	0.680	2.63 (0.09)	2.83 (0.09)	−1.61	0.109	2.73 (0.06)
***Emotional Outburst***									
I yelled, screamed, or got mad	1.77 (0.09)	2.01 (0.09)	1.96	0.052	1.84 (0.09)	1.94 (0.09)	−0.80	0.426	1.89 (0.06)
***Self-Blame***									
I blamed myself for causing the problem	2.44 (0.09)	2.76 (0.09)	2.40	0.017 *	2.65 (0.09)	2.54 (0.10)	0.87	0.386	2.59 (0.07)
***Blaming Others***									
I blamed someone else for causing the problem	2.07 (0.09)	1.84 (0.08)	−1.83	0.068	2.01 (0.09)	1.90 (0.09)	0.88	0.378	1.96 (0.06)
***Wishful Thinking***									
I wished the problem had never happened	3.32 (0.08)	3.51 (0.07)	1.79	0.075	3.49 (0.07)	3.34 (0.08)	1.47	0.142	3.42 (0.05)
I wished I could make things different	3.11 (0.08)	3.25 (0.07)	1.31	0.191	3.14 (0.08)	3.21 (0.07)	−0.62	0.536	3.18 (0.05)
***Resignation***									
I didn’t do anything because the problem couldn’t be fixed	2.35 (0.10)	2.41 (0.09)	0.46	0.647	2.41(0.10)	2.34 (0.09)	0.55	0.581	2.38 (0.07)
**Control-Oriented**									
***Distraction***									
I just tried to forget it	2.89 (0.09)	2.86 (0.08)	−0.28	0.782	2.93 (0.08)	2.88 (0.06)	0.88	0.380	2.88 (0.06)
I did something like played a game to forget it	3.05 (0.91)	2.90 (0.09)	−1.32	0.188	3.04 (0.08)	2.91 (0.06)	1.07	0.287	2.97 (0.06)
***Cognitive Restructuring***									
I tried to see the good side of things	3.00 (0.08)	3.04 (0.08)	0.32	0.747	3.09 (0.08)	2.94 (0.09)	1.34	0.182	3.02 (0.06)
***Problem-Solving***									
I tried to fix the problem by thinking of answers	3.10 (0.08)	3.21 (0.07)	0.97	0.334	3.23 (0.07)	3.07 (0.08)	1.44	0.152	3.15 (0.06)
I tried to fix the problem by doing something or talking to someone	2.85 (0.08)	2.94 (0.08)	0.79	0.432	2.99 (0.08)	2.79 (0.08)	1.71	0.088	2.89 (0.06)
***Emotional Regulation***									
I tried to calm myself down	3.04 (0.79)	3.14 (0.07)	0.96	0.340	3.10 (0.07)	3.07 (0.08)	0.26	0.794	3.09 (0.05)
***Social Support***									
I tried to feel better by spending time with other family or friends	3.35 (0.07)	3.36 (0.07)	0.17	0.862	3.49 (0.06)	3.21 (0.08)	2.79	0.003 **	3.35 (0.05)

Note. * significant at *p* ≤ 0.05; ** significant at *p* ≤ 0.01; t = test statistic.

**Table 3:** Multivariable regressions on measured dimensions of the SDQ.

		Emotional Symptoms			Conduct Problems			Hyperactivity			Peer Problems		
Model		β	SE	*p*	β	SE	*p*	β	SE	*p*	β	SE	*p*
M1													
	Age	−0.16	0.10	0.106	0.14	0.08	0.087	0.25	0.09	0.006 **	0.16	0.09	0.078
	Sex	−1.88	0.27	0.000 ***	−0.34	0.22	0.114	−0.01	0.25	0.994	−0.25	0.24	0.287
M2													
	Age	−0.20	0.11	0.062	0.09	0.08	0.270	0.19	0.09	0.035	0.11	0.09	0.206
	Sex	−1.90	0.27	0.000 ***	−0.36	0.21	0.085	−0.03	0.24	0.912	−0.15	0.23	0.506
	Control-Oriented	−0.43	−0.91	0.365	−1.45	0.35	0.000 ***	−1.54	0.35	0.000 ***	−1.22	0.42	0.003 **
	Escape-Oriented	3.24	0.50	0.000	0.52	0.41	0.207	0.93	0.48	0.053	1.56	0.49	0.002
M3													
	Age	−0.19	0.10	0.059	0.07	0.08	0.399	0.16	0.10	0.087	0.06	0.08	0.497
	Sex	−1.60	0.26	0.000 ***	−0.38	0.20	0.063	−0.01	0.24	0.969	−0.23	0.21	0.287
	Control-Oriented	−0.24	0.52	0.649	−1.11	0.40	0.006 **	−1.28	0.38	0.001 **	−0.22	0.39	0.574
	Escape-Oriented	3.19	0.50	0.000 ***	−0.44	0.44	0.323	0.79	0.49	0.108	1.43	0.48	0.003 **
	Friends	−0.01	0.03	0.746	−0.03	0.03	0.323	0.01	0.03	0.786	−0.09	0.03	0.001 **
	Family	−0.03	0.03	0.450	−0.04	0.03	0.199	−0.06	0.03	0.088	−0.08	0.03	0.004 **
	Significant Others	−0.01	0.03	0.824	0.01	0.03	0.812	0.01	0.03	0.834	−0.02	0.03	0.482

Note. Emotional Systems: R^2^ = 0.16; *p* = 0.000 for M1; R^2^ = 0.27; *p* ≤ 0.001 for M2; R^2^ = 0.28; *p* = 0.791 for M3. Conduct Problems: R^2^ = 0.02; *p* = 0.045 for M1; R^2^ = 0.09; *p* = 0.002 for M2, R^2^ = 0.11; *p* = 0.383 for M3. Hyperactivity: R^2^ = 0.03; *p* = 0.0198 for M1; R^2^ = 0.09; *p* = 0.001 for M2; R^2^ = 0.10; *p* = 0.394 for M3. Peer Problems: R^2^ = 0.02; *p* ≤ 0.138 in M1; R^2^ = 0.083; *p* = 0.000 in M2; R^2^ = 0.21; *p* ≤ 0.001 in M3. ** significant at *p* ≤ 0.01; *** significant at *p* ≤ 0.001.

**Table 4:** Multivariable regressions on mental wellbeing (WHO-5), prosocial behavior (SDQ), and resilience (CYRM).

		Mental Wellbeing (WHO-5 Wellbeing Index)			Prosocial Behavior (SDQ Subscale)			Resilience (CYRM-R)		
Model		β	SE	*p*	β	SE	*p*	β	SE	*p*
M1										
	Age	−1.90	1.05	0.070	−0.21	0.08	0.012 *	−1.53	0.36	0.000 ***
	Sex	4.66	2.67	0.081	−0.22	0.23	0.345	−0.14	0.98	0.844
M2										
	Age	−1.08	0.99	0.280	−0.14	0.08	0.060	−1.12	0.34	0.001 **
	Sex	5.00	2.56	0.052	−0.05	0.21	0.805	0.19	0.87	0.828
	Control-Oriented	22.66	4.04	0.000 ***	1.94	0.34	0.000 **	11.34	1.53	0.000 ***
	Escape-Oriented	−6.38	5.27	0.227	0.87	0.43	0.046 *	−1.46	1.76	0.406
M3										
	Age	−0.10	0.95	0.919	−0.07	0.07	0.373	−0.50	0.30	0.099
	Sex	5.89	2.33	0.012 **	−0.01	0.20	0.970	0.67	0.64	0.299
	Control-Oriented	11.59	3.96	0.004 **	0.50	0.14	0.001 **	4.49	1.22	0.000 ***
	Escape-Oriented	−5.12	4.73	0.280	0.47	0.15	0.019 *	0.04	1.29	0.976
	Friends	0.75	0.28	0.007 **	0.04	0.03	0.216	0.36	0.09	0.000 ***
	Family	0.85	0.29	0.004 **	0.08	0.03	0.004 **	0.77	0.09	0.000 ***
	Significant Others	0.81	0.29	0.005 **	0.04	0.03	0.117	0.25	0.09	0.006 **

Note. Mental Wellbeing: R^2^ = 0.03; *p* = 0.350 for M1; R^2^ = 0.14; *p* < 0.001 for M2; R^2^ = 0.29; *p* ≤ 0.000 for M3. Prosocial Behavior: R^2^ = 0.02; *p* = 0.034 for M1; R^2^ = 0.17; *p* ≤ 0.001 for M2; R^2^ = 0.25; *p* ≤ 0.001 for M3. Resilience: R^2^ = 0.06; *p* < 0.001 for M1; R^2^ = 0.27; *p* ≤ 0.001 for M2; R^2^ = 0.58; *p* ≤ 0.001 for M3. * significant at *p* ≤ 0.05; ** significant at *p* ≤ 0.01; *** significant at *p* ≤ 0.001.

**Table 5:** Path analysis summary.

Relationship	Total Effect	Direct Effect	Indirect Effect	Confidence Interval		t-Statistic	*p*-Value	Conclusion
				Lower Bound	Upper Bound			
Social Support -> Control-Oriented Coping -> Total Difficulties	−0.13	−0.10	−0.03	−0.05	−0.00	−2.00	0.046	Supports partial mediation
Social Support -> Control-Oriented Coping -> Total Resilience	0.48	0.42	0.07	0.04	0.10	4.29	<0.001	Supports partial mediation

## Data Availability

The data presented in this study are available on request from the corresponding author to ensure data safety.
